# Plasma retinol, carotene and vitamin E concentrations and lung function in a crocidolite-exposed cohort from Wittenoom, Western Australia: a cohort study

**DOI:** 10.1186/1475-2891-4-16

**Published:** 2005-05-11

**Authors:** Helman S Alfonso, Lin Fritschi, Nicholas H de Klerk, Gina Ambrosini, John Beilby, Nola Olsen, A William Musk

**Affiliations:** 1 School of Population Health, University of Western Australia, Perth, Western Australia, Australia; 2 School of Public Health, Curtin University of Technology, Perth, Western Australia; 3 Department of Respiratory Medicine, Sir Charles Gairdner Hospital, Perth, Western Australia, Australia; 4 Department of Biostatistics and Genetic Epidemiology, Telethon Institute for Child Health Research, Perth, Western Australia, Australia; 5 Clinical Biochemistry, PathCentre, Western Australia

## Abstract

**Background:**

Increased rates of death from asbestos related diseases have been reported for people previously employed in the mining and milling operations at Wittenoom (Western Australia), and people who lived in the nearby town, where they were environmentally exposed to crocidolite.

**Methods:**

Annual measurements of forced expiratory volume in 1 second (FEV1) and forced vital capacity (FVC) and plasma concentrations of retinol, carotene and vitamin E have been made since 1992. Mixed effects models were used to examine the associations between lung function and the plasma vitamin levels of retinol, carotene and vitamin E.

**Results:**

After adjusting for potential confounders, higher plasma retinol and carotene concentrations were significantly associated with higher levels of lung function at entry into the study, while vitamin E concentrations were associated with lower entry lung function. Retinol was associated with a less steep decline of lung function over time, while carotene concentrations were associated with an increased decline of lung function over time and vitamin E levels were not associated with changes of lung function over time.

**Conclusion:**

These results support a beneficial relationship between plasma concentrations of retinol on the levels and rates of change of lung function, while showing no such consistent beneficial effect for plasma levels of beta-carotene or vitamin E.

## Introduction

Respiratory impairment is an important cause of disability in asbestos-exposed people. Cross-sectional and longitudinal studies have shown that greater intensity and duration of asbestos exposure are associated with greater impairment of pulmonary function [1]. A protective effect of fruit and vegetable consumption on lung function has been reported, and a number of studies have found positive associations between pulmonary function and dietary intake and blood levels of both carotenoids and retinoids [2-4].

Crocidolite (blue asbestos) was mined at Wittenoom, Western Australia from 1943 until 1966. Approximately 7000 workers were employed in the mining and milling operations [5]. In addition, about 5000 people, who did not work in the production of asbestos, are documented to have lived in the nearby town, where they received environmental exposure to asbestos [6]. Former workers and residents were also frequently cigarette smokers, and increased risks of asbestos- and smoking-related diseases have been shown in these populations [6].

Cohorts of former workers and residents of Wittenoom have been followed up continuously since 1975, and since 1990 some of them have participated in an intervention program including supplemental vitamin A in an attempt to reduce the occurrence of malignant mesothelioma and lung cancer [7,8]. The Program also included advice on diet, stopping smoking and physical exercise. As no beneficial effect was reported for beta-carotene after five years [7,8], and other studies showed increased risks of developing lung cancer in subjects taking beta-carotene supplements [9,10], all participants have been provided with retinol since 1997.

The aim of this analysis was to examine the relationships between lung function and plasma levels of retinol, carotene and vitamin E, after controlling for potential confounders, in people exposed to crocidolite in Wittenoom who have participated in the Vitamin A Program. In analysing the relationships between plasma vitamin concentrations and lung function, we are not intending to assess of the efficacy of the Program, which will be described in a separate report (Musk *et al*, in preparation). The predictors of the levels and the rate of change of lung function, as well as the assessment of the efficacy of the Program, have been described in separate reports ([11,33]).

### Subjects

Cohorts of former workers and residents of Wittenoom have been followed up continuously since 1975. In 1989, all ex-residents or ex-workers that could be located were invited to participate in the Vitamin A Program, and the Program has commenced accepting in July 1990. Each participant attends a clinic annually to receive a year's supply of the vitamin supplement. Annual spirometric measurements and plasma vitamins determinations have also been taken since 1992. All participants who had at least one spirometric measurement and a blood test for plasma vitamin concentrations performed on the same day were included in the present study. Participants were followed from the time of their first test, until the last result available before 23 September 2002. People younger than 25 years old were excluded from the analysis, because growth in lung function continues until this age [12].

## Methods

### Plasma vitamin concentrations

On the same day as the spirometry was performed, a non-fasting blood sample was collected and the plasma concentrations of retinol, total carotene, alpha-carotene, beta-carotene and alpha-tocopherol (vitamin E) were determined by reverse-phase high performance liquid chromatography (HPLC) [13]. Total carotene was measured from July 1990 to May 1994, but subsequently, alpha- and beta-carotene were measured separately. For this analysis, after May 1994, total carotene was assumed to be the sum of alpha-carotene and beta-carotene. For measurements whose plasma values were undetectable by laboratory methods, a value midpoint between zero and the laboratory's minimum detectable value was used (42% in alpha-carotene measurements, and 19% in beta-carotene) [14,33].

### Pulmonary Function Testing

FEV_1 _and FVC measurements were performed by trained technicians according to the guidelines of the American Thoracic Society (ATS) [15] and adjusted for body temperature and pressure-saturated with water vapour. The highest of at least three technically satisfactory attempts was selected. Only measurements in which the two highest attempts fulfilled the ATS criteria for reproducibility (an agreement within 5%) were included in the analysis.

### Asbestos Exposure Assessment

Category of exposure (former worker or resident of Wittenoom), age at first exposure, cumulative asbestos exposure and radiographic asbestosis at first visit were the indicators of crocidolite exposure used in the present analysis. The three first indicators were obtained from the Vitamin A Program records.

Methods describing the assessment of cumulative asbestos exposure for workers have been described previously [16,33]: job histories were obtained from employment records, and fibre concentrations for all job categories were estimated from the results of a survey of airborne respirable fibres of crocidolite that was carried out at various work sites at Wittenoom in 1966, and particle counts performed by the Mines Department of Western Australia throughout the period of operation of the mining industry. Each subject's cumulative exposure was calculated by adding over all their different jobs the product of fibre concentration and the length of time in that job.

For ex-residents, the estimation of individual asbestos exposure levels has also been described in detail elsewhere [6]. Subjects not working directly with asbestos were assigned an intensity of exposure of 1 fibre/millilitre (f/ml) from 1943 to 1957, when a new mill was commissioned and the town was moved, and then 0.5 f/ml between 1958 and 1966, when the mining operation ceased. Since then, interpolation between periodic hygiene surveys using personal monitors assigned exposures from 0.5 f/ml in 1966 to 0.01 f/ml in 1992. Duration of residence was obtained from a questionnaire completed at the time of attending the Vitamin A Program. Individual cumulative exposure was calculated by combining the duration of residence and the intensity of exposure for each person. These calculations have been demonstrated to be internally valid on the basis of lung fibre determinations and dose-response characteristics for mesothelioma, lung cancer and asbestosis [6].

To assess radiographic asbestosis, without knowledge of exposure status, a panel of trained and experienced readers read the plain chest radiograph at the first visit according to the International Labour Office's (ILO) classification of the chest x-ray manifestations of pneumoconioses [17], utilizing the standard films provided by the ILO for side by side comparison. For the purpose of this study, radiographic asbestosis was defined as a profusion score of 1/0 or greater, as indicated in the ILO guidelines [17].

### Smoking History Assessment

Smoking history was obtained from a questionnaire administered at entry to the Program. Information on smoking status (never-smokers, ex-smokers and current smokers) was evaluated as a categorical variable. The history over time of smoking was not available for this analysis.

### Statistical Analysis

To assess the relationships between lung function and plasma vitamin concentrations, the dependent variables (FEV_1 _and FVC) were regressed on time, controlling for demographic characteristics (sex, age and height), asbestos exposure and tobacco smoking. Plasma levels of retinol, carotene and vitamin E (as quartiles) were included in the model, both separately and at the same time. A linear trend was also evaluated by including the raw values (continuos variable) of each plasma vitamin concentration. The main effect of each vitamin concentration was interpreted as the level of lung function at entry into the study, while the interaction of each vitamin concentration with time of follow up (in years) was interpreted as the annual change of lung function over time [18]. Specifically, levels of lung function at entry into the Program correspond to the values of lung function (or differences between groups) when time of follow up was zero. The analyses were performed initially for each vitamin separately, and then by including simultaneously the three vitamin values into the model.

The data were modelled by a general linear mixed-effects model, using SAS PROC MIXED [19]. Random-effects models are most appropriate for unbalanced longitudinal data or when the intervals between measurements for each subject are not equally spaced [18]. The model-building process was performed following the steps suggested by Verbeke and Molenberghs [18]. Initially, a model with all the explanatory variables and their biologically plausible interactions was applied in order to remove any systematic trends. In the second step, random effects were included in the model; the best model was selected according to a likelihood ratio test. Thirdly, several residual covariance structures were tested and the best was selected according to the Akaike Information Criterion (AIC). Finally, the model was simplified by deleting non-significant terms. Estimation was made by the restricted maximum likelihood method, and tests were performed using the 5% level of significance. Model validation was carried out by checking normal distribution of residuals.

## Results

A total of 5,750 determinations from 1,378 subjects who had at least one test of both spirometry and plasma vitamin concentrations were analysed. The demographic characteristics and exposure histories at the first visit showed that former workers had lower levels of FEV_1 _and FVC than ex-residents (Table 1). Workers were more likely to be male and residents were female. Workers were exposed to higher cumulative amounts of asbestos (median 6.40 fibres/ml per year, interquartile range 1.92–26.01), and included a higher proportion of participants with radiographic asbestosis. Although ex-residents were exposed to asbestos for longer periods of time, their cumulative asbestos exposure was lower, as the intensity of exposure was much higher in the mining and milling processes. Ex-residents tended to be exposed to asbestos at a younger age than workers. In addition, workers included a higher proportion of ever-smokers. Initial levels of plasma vitamins were similar between workers and ex-residents. At the first visit, plasma retinol concentrations were not significantly correlated with plasma carotene concentrations (r = -0.006, p = 0.84); plasma concentrations of retinol and vitamin E were highly correlated (r = 0.33, p = <0.0001); and carotene and vitamin E concentrations tended to be correlated (r = 0.047, p = 0.08).

**Table 1:** Demographic characteristics of participants at first visit ^a^

	**Residents**	**Workers**
Participants, n (%)	567 (41.1)	811 (58.9)
Male, n (%)	270 (47.6)	749 (92.4)
FEV_1_, litres ^a^	2.9 (0.9)	2.7 (0.7)
FVC, litres ^a^	3.7 (1.1)	3.6 (0.9)
Age, yr ^a^	50.8 (12.5)	59.2 (7.7)
Height at baseline, cm ^a^	168 (9.5)	171 (7.5)
Cumulative asbestos, f/ml-year ^a^	6.9 (7.5)	24.5 (47.8)
Age at first asbestos exposure, yr ^a^	13.9 (12.7)	24.8 (6.1)
Radiographic asbestosis, n (%)	8 (1.4)	143 (17.6)
Current-smokers, n (%)	114 (19.6)	182 (21.8)
Ex-smokers, n (%)	183 (31.5)	452 (54.2)
Never-smokers, n (%)	284 (48.9)	199 (23.9)
Plasma retinol, (μmol/L)^a^	2.70 (0.70)	2.70 (0.69)
Plasma carotene, (μmol/L)^a^	1.38 (1.57)	1.38 (1.52)
Plasma vitamin E, (μmol/L)^a^	38.13 (13.85)	35.43 (11.40)

^a^mean (SD) range

Twenty-four percent of participants had one visit at which lung function and plasma vitamins were measured, 21% had two measurements, 17% had three, 14% had four, 10% had five, and 14% had more than 5 measurements during the period of observation. The Vitamin A Program initially gave preference to enrolling workers, who were mostly males, because of their greater risks of developing asbestos-related diseases. Therefore they had more spirometric measurements per person and a longer follow-up time (Table 2).

**Table 2:** Characteristics of the follow up of the study cohort ^a^

	**Residents**	**Workers**
Total lung function and blood tests, n (%)	1942 (33.7)	3898 (66.2)
Measurements per person, ^a^	3.43 (1.5)	4.7 (2.5)
Follow up, years, ^a^	3.0 (1.7)	5.4 (3.3)
Months between measurements,^a^	14.0 (4.8)	16.5 (9.7)

^a^mean (SD

After adjusting for confounders, plasma retinol concentrations (as a single vitamin in the model) at entry into the study were not associated with levels of lung function (Table 3), (p-value for linear trend = 0.36 for FEV1, p = 0.50 for FVC). For example, people in the third quartile (Q3) have, on average, 4.4 ml more of FEV1 and 6.6 ml more of FVC, compared to those in the first quartile (Q1). However, these differences were not statistically significant as the confidence intervals contain negative values. However, higher concentrations of plasma retinol were associated with a lower rate of decrease in lung function over time: the annual decline in people in the highest quartile of retinol concentration was 11.3 ml (95% CI = 4.8–17.7) of FEV1 and 18.6 ml (95% CI = 10.4–26.8) of FVC less steeper than those in the lowest quartile. The linear trends were significant for FEV1 and FVC (not shown in table 3).

**Table 3:** Relationships between plasma retinol concentrations and lung function levels and annual rate of change*

	Quartiles	FEV1	95%CI	FVC	95%CI
Level at entry into program (ml)	Q1	0		0	
	Q2	5.5	-15.5,26.6	-8.9	-35.8,17.9
	Q3	4.4	-19.8,28.7	6.6	-24.4,37.6
	Q4	2.6	-25.5,30.9	-16.8	-52.7,19.1

Annual change (ml/year)	Q1	0		0	0
	Q2	4.1	-1.8,10.1	10.3	2.7,17.9
	Q3	7.1	1.0,13.4	11.1	3.2,19.1
	Q4	11.3	4.8,17.7	18.6	2.7,10.3

*Adjusted for age, sex, height, asbestos exposure and smoking history

Quartile 1: Retinol concentrations lower than 2.5 μmol/L; Quartile 2: concentrations more than 2.5 and less than 3.0 μmol/L; quartile 3: concentrations more than 3.0 and less than 3.5; and quartile 4: concentrations equal or greater than 3.5 μmol/L.

Higher plasma carotene concentrations (as a single vitamin in the model) were associated with higher levels of lung function at entry into the study (Table 4). On the contrary, higher plasma carotene concentrations were associated with a steeper decline of lung function. The annual decline in people in the highest quartile was 9.3 ml (95% CI = 2.6–15.9) of FEV1, and 8.3 ml (95% CI = 0–16.5) of FVC less steeper than those in the lowest quartile (Table 4). Linear trends were significant for both levels and rates of change of lung function (not shown).

**Table 4:** Relationships between plasma carotene concentrations and lung function levels and annual rate of change*

	Quartiles	FEV1	95%CI	FVC	95%CI
Level at entry into program (ml)	Q1	0		0	
	Q2	21.4	-3.2,46.1	0.06	-31.4,31.6
	Q3	47.8	19.1,76.4	30.6	-5.8,67.0
	Q4	67.3	38.0,96.6	59.5	22.6,96.5

Annual change (ml/year)	Q1	0	0	0	0
	Q2	-1.1	-6.5,4.3	3.04	-3.8-9.9
	Q3	-6.0	-12.3,0.3	-6.6	-14.6-1.5
	Q4	-9.3	-16.0,-2.7	-8.3	-16.8-0.1

Adjusted for age, sex, height, asbestos exposure and smoking history.

Quartile 1: Carotene concentrations lower than 0.45 μmol/L; Quartile 2: concentrations more than 0.45 and less than 0.8 μmol/L; quartile 3: concentrations more than 0.8 and less than 1.3; and quartile 4: concentrations equal or greater than 1.3 μmol/L.

Higher plasma vitamin E concentrations (as a single vitamin in the model) were associated with higher levels of lung function at entry into the study (Table 4). However, higher plasma vitamin E concentrations were associated with a steeper decline of lung function. The decline in people in the highest quartile was 7.9 ml (95% CI = 14.3-1.6) per year in FEV1, and 13.9 ml (95% CI = 21.9-6.0) per year in FVC, compared to those in the lowest quartile (Table 4). Linear trends were significant for both levels and rates of change of lung function (not shown).

Including retinol, carotene and vitamin E plasma concentrations in the same model, and adjusting for potential confounders, higher plasma retinol concentrations were associated with higher levels of lung function, as well as a slower decline in the annual rate of change of lung function (Table 6). Plasma carotene concentrations were associated with higher levels of lung function at entry, but with a steeper decline in lung function over time. Plasma vitamin E concentrations were associated with lower levels of lung function at entry into the study, but not associated with changes over time.

**Table 6:** Relationships between lung function and retinol, carotene and vitamin E concentrations, when the three vitamins were simultaneously included in the model*

Plasma vitamin concentrations (μMol/L)		FEV1 (ml) Estimate (SD)	p-value	FVC (ml) Estimate (SD)	p-value
Retinol	Level	13.9 (7.5)	0.06	16.6 (9.5)	0.08
	Change	3.5 (1.5)	0.02	5.7 (1.9)	0.00
Carotene	Level	14.6 (3.6)	0.00	13.1 (4.5)	0.00
	Change	-2.6 (1.0)	0.01	-3.5 (1.3)	0.01
Vitamin E	Level	-1.0 (0.4)	0.01	-2.0 (0.5)	<0.01
	Change	0.07 (0.09)	0.38	0.2 (0.1)	0.13

* Adjusted for age, sex, height, asbestos exposure and smoking history. Only results for linear trend are shown.

The analysis was also performed for alpha- and beta-carotene. As was reported for total carotene concentrations, both alpha- and beta-carotene concentrations were associated with higher levels of lung function, and inversely related to the change of lung function over time (not shown).

## Discussion

After adjusting for potential confounders and the joint effect of the plasma vitamin levels, plasma retinol concentrations were associated with higher levels of lung function as well as a slower rate of decline over time; plasma carotene concentrations (or alpha and beta-carotene) were associated with higher levels of lung function, but with a steeper decline in lung function; plasma vitamin E concentrations were associated with lower levels of lung function at entry, but not associated with amount change of lung function over time. The observed tendency in the separate analysis for each vitamin (Tables 3 to 5) was similar to the results obtained when the three variables were jointly included in the model, except for the effect of plasma retinol concentrations, which became positively associated with levels of lung function at the entry in the Vitamin A Program. We have previously shown that mortality in subjects with asbestosis was inversley related to plasma concentrations of retinol and vitamin E (at first visit and during the follow up period),  while carotene concentrations at the first visit were associated with lower mortality but not during the follow up period [33]

**Table 5:** Relationships between plasma vitamin E concentrations and lung function levels and annual rate of change*

	Quartiles	FEV1	95%CI	FVC	95%CI
Level at entry into program (ml)	Q1	0		0	
	Q2	23.5	-1.9, 49.0	33.5	1.0, 66.0
	Q3	38.4	12.0, 64.8	67.0	33.4, 100.6
	Q4	35.9	8.1, 63.7	82.3	47.1, 117.5

Annual change (ml/year)	Q1	0		0	
	Q2	-6.8	-10.1, 0.9	-4.4	-11.4, 2.7
	Q3	-4.6	-12.7, -1.0	-7.8	-15.3, -0.3
	Q4	-7.9	-14.3, -1.6	-13.9	-21.9, -6.0

Adjusted for age, sex, height, asbestos exposure and smoking history.

Quartile 1: Vitamin E concentrations lower than 31 μmol/L; Quartile 2: concentrations more than 31 and less than 37 μmol/L; quartile 3: concentrations more than 37 and less than 45; and quartile 4: concentrations equal or greater than 45 μmol/L.

The positive effect of plasma retinol on lung function has been recognised in previous cross-sectional and prospective studies [3,4], but not in longitudinal studies with multiple measurements per participant. This positive effect is consistent with the multiple biologic functions of retinoids, including regulation of cell proliferation, cell differentiation and morphogenesis [20]. In contrast to carotenoids, retinol is not a potent free radical scavenger; therefore other mechanisms have been advocated for explaining its possible beneficial effects [2]. For example, retinoids have a variety of effects on the cell membrane, involving altered glycoprotein synthesis and changes in membrane receptors for various hormones, including those mediated by c-AMP. The actions on these receptors may influence cell-cell interactions, cell adhesion, and cell membrane permeability. In a recent animal model, systemic administration of *all-trans-*retinoic acid (ATRA) reversed manifestations of elastase-induced emphysema [21]. Similar results have now been replicated by Belloni and colleagues with both ATRA and 9-*cis*-retinoic acid [22]. This is a promising finding, although is not possible to draw a close parallel between an elastase-induced emphysema murine model and the pathogenesis of human chronic obstructive pulmonary disease.

The positive association between blood carotene concentrations and the levels of lung function has also been previously recognized in cross-sectional and prospective studies [3,23,24]. The biological plausibility for a protective effect of carotenoids on lung function is associated with the multiple biological actions of these micronutrients, in particular their role in scavenging oxidised products [2]. It has been postulated that antioxidant defences play a critical role in the respiratory tract where high exposure to endogenous and environmental oxidants occurs, which may impair ventilatory function by inducing inflammation and destruction of the alveolar walls [25]. However, our results showed that plasma carotene concentrations were associated with a steeper decline of lung function. This disappointing effect mirrors the results obtained in this Vitamin A Program and other intervention studies, in which higher-than-expected rates of lung cancer were found in people taking beta-carotene [9,10].

Several observational studies have consistently showed that individuals who eat more fruits and vegetables tend to have lower risk of developing various cancers and other chronic diseases [26]. Several studies have found positive associations between pulmonary function and dietary intake and blood levels of both carotenoids and retinoids [2]. From the many potential nutrients in fruits and vegetables, beta-carotene was credited as the most important nutrient likely to be responsible for this beneficial effect, especially following the influential paper from Peto and colleagues [27]. However, results from at least 8 trials have shown that beta-carotene supplementation has essentially no significant role in decreasing cancer risk [28]. No previous studies have been reported on relationships between supplemental beta-carotene and lung function.

Two alternative explanations have been proposed to explain the conflicting results between observational and intervention studies: first, that beta-carotene is not the factor responsible for the beneficial effect of fruit and vegetable intake on cancer [28]; second, that the supplemental synthetic *all-trans*-beta-carotene does not reproduce the effect of the natural counterpart [29].

It is possible that many of the studies suggesting that dietary beta-carotene is protective were confounded [28]. Observational studies do not permit causal relationships to be established. Hundreds of carotenoids and thousands of nutrients exist in foods. Researchers are only able to examine and study a few hundred of them, and the synergy among these numerous nutrients may play a larger role that any single compound [28]. Although the reasons remain unclear, current wisdom indicates no role for synthetic beta-carotene supplementation for preventing cancer or improving lung function.

Despite being considered a powerful anti-oxidant, we did not find a consistent effect of Vitamin E on lung function. Whether the inverse relationships reported in the univariate analysis of this study reflects real relations peculiar to the subjects studied, or are the result of sampling variation or chance may be clarified by future studies.

Due to self-selection into the Vitamin A Program this population may not be representative of the whole cohort exposed to crocidolite at Wittenoom. People who participated in the Program were younger and had higher cumulative asbestos exposure [7]. The results from this study, may not apply to subjects with lower exposure to crocidolite or older age groups.

An additional difficulty in interpreting the results of our study may be that we examined vitamin levels in a population in which the vitamin levels were artificially increased and did not adjust for their assigned dose. Although the arm of the study to which participants had originally been assigned was known, we had no precise way of measuring their actual intake of vitamins, nor their dietary intake of fruit and vegetables. However, epidemiological theory [30] postulates a hierarchy of exposure measurement from available dose in the general environment (assigned vitamins), to administered dose (number of tablets taken), to absorbed dose (dose taken up from the gastrointestinal tract), to active dose at different target sites. The closer that the measurement is to the active dose, the more likely it is that a study will disclose any real relationship. Since we were able to measure the serum level of vitamins, adjusting for the assigned dose does not make theoretical sense, and would possibly artifactually decrease any relationship observed.

One problem in longitudinal studies is the occurrence of missing data due to withdrawals, which may result in follow-up bias [31]. Ninety subjects (6.3% of all participants) dropped out of the study, and 142 participants who during the follow up period. The people who died were not counted as dropouts as they had complete data [18]. People who abandoned the Program had lower levels of FEV_1_, FVC and FEV_1 _/FVC, compared to those who remained. We have assumed a 'missing at random pattern', implying that any withdrawal from the study did not depend on the level of lung function itself, although it may depend on some covariates. With a missing at random pattern, methods based on likelihood will produce valid inferences, if the model specification is correct [32]. Applying an available case analysis, using PROC MIXED of SAS, the reported relationships may be interpreted as the response conditional on the subject remaining in the study [18].

The present study has the advantage of having both cross-sectional and longitudinal components in the analysis of the data, which make efficient use of all the available information and explores hypotheses unable to be tested with cross-sectional approaches alone [33]. However, the accurate evaluation of tobacco smoke exposure in this report, as in most others, has some limitations because it was based on self-reporting. This report analyses the smoking status at the beginning of the follow up, and does not include the analysis of changes in smoking habits over time.

This longitudinal study demonstrates a beneficial relationship between plasma levels of retinol and level and rate of decline of lung function as measure by FEV1 and FVC, while showing no such beneficial effect for plasma levels of beta-carotene.

## Ethical Approval

All subjects gave their informed consent and the study was approved by the Human Research Ethics Committee of the University of Western Australia and the Clinical Drug Trials Committee of the Sir Charles Gairdner Hospital, Nedlands, Western Australia.

## References

[B1] Rom WN (1992). Accelerated loss of lung function and alveolitis in a longitudinal study of non-smoking individuals with occupational exposure to asbestos. American Journal of Industrial Medicine.

[B2] Schunemann HJ, Freudenheim JL, Grant BJ (2001). Epidemiologic evidence linking antioxidant vitamins to pulmonary function and airway obstruction. Epidemiol Rev.

[B3] Chuwers P, Barnhart S, Blanc P, Brodkin CA, Cullen M, Kelly T, Keogh J, Omenn GS, Williams J, Balmes JR (1997). The protective effect of beta-carotene and retinol on ventilatory function in an asbestos-exposed cohort. American Journal of Respiratory and Critical Care Medicine.

[B4] Grievink L, Smit HA, Veer P, Brunekreef B, Kromhout D (1999). Plasma concentrations of the antioxidants beta-carotene and alpha-tocopherol in relation to lung function. European Journal of Clinical Nutrition.

[B5] Musk AW, de Klerk NH, Eccles JL, Hobbs MS, Armstrong BK, Layman L, McNulty JC (1992). Wittenoom, Western Australia: A modern industrial disaster. American Journal of Industrial Medicine.

[B6] Hansen J, de Klerk NH, Musk AW, Hobbs MS (1998). Environmental exposure to crocidolite and mesothelioma: exposure-response relationships. American Journal of Respiratory and Critical Care Medicine.

[B7] Musk AW, de Klerk NH, Ambrosini GL, Eccles JL, Hansen J, Olsen NJ, Watts VL, Lund HG, Pang SC, Beilby J, Hobbs MS (1998). Vitamin A and cancer prevention I: observations in workers previously exposed to asbestos at Wittenoom, Western Australia. International Journal of Cancer.

[B8] de Klerk NH, Musk AW, Ambrosini GL, Eccles JL, Hansen J, Olsen N, Watts VL, Lund HG, Pang SC, Beilby J, Hobbs MS (1998). Vitamin A and cancer prevention II: comparison of the effects of retinol and beta-carotene. International Journal of Cancer.

[B9] Omenn GS, Goodman GE, Thornquist MD, Balmes J, Cullen MR, Glass A, Keogh JP, Meyskens FL, Valanis B, Williams JH, Barnhart S, Hammar S (1996). Effects of a combination of beta carotene and vitamin A on lung cancer and cardiovascular disease.. New England Journal of Medicine.

[B10] ATBCCPSG (1994). The effect of vitamin E and beta carotene on the incidence of lung cancer and other cancers in male smokers.  The Alpha-Tocopherol, Beta Carotene Cancer Prevention Study Group.. New England Journal of Medicine.

[B11] Alfonso HS, Fritschi L, de Klerk NH, Olsen N, Sleith J, Musk AW (2004). Effects of asbestos and smoking on the levels and rates of change of lung function in a crocidolite exposed cohort in Western Australia. Thorax.

[B12] Quanjer PH, Tammeling GJ, Cotes JE, Pedersen OF, Peslin R, Yernault JC (1993). Lung volumes and forced ventilatory flows. Report Working Party Standardization of Lung Function Tests, European Community for Steel and Coal. Official Statement of the European Respiratory Society. European Respiratory Journal.

[B13] Milne DB, Botnen J (1986). Retinol, alpha-tocopherol, lycopene, and alpha- and beta-carotene simultaneously determined in plasma by isocratic liquid chromatography. Clinical Chemistry.

[B14] Clarke JU (1998). Evaluation of Censored Data Methods to Allow Statistical Comparisons Among Very Small Samples with Below Detection Limit Observations. Environmental Science & Technology.

[B15] American Thoracic Society (1987). Standardization of spirometry--1987 update. Statement of the American Thoracic Society. American Review of Respiratory Disease.

[B16] de Klerk NH, Musk AW, Armstrong BK, Hobbs MS (1991). Smoking, exposure to crocidolite, and the incidence of lung cancer and asbestosis. British Journal of Industrial Medicine.

[B17] International Labour Organization (1980). International classification of radiographs of pneumoconiosis. Guidelines for the use International Labor Organization (ILO). Geneva: International Labor Office..

[B18] Verbeke G, Molenberghs G (2000). Linear mixed models for longitudinal analysis.

[B19] SAS/STAT (1999). User's guide, version 8.2.

[B20] Yoshimi T, Takahashi Y, Takahashi S, Miura T (2000). Changes in lung-specific molecular expression during differentiation of hamster embryonic M3E3/C3 cell line. Biochemistry and Cell Biology.

[B21] Massaro GD, Massaro D (2000). Retinoic acid treatment partially rescues failed septation in rats and in mice. American Journal of Physiology.

[B22] Belloni PN, Garvin L, Mao CP, Bailey-Healy I, Leaffer D (2000). Effects of all-trans-retinoic acid in promoting alveolar repair. Chest.

[B23] Schunemann HJ, Grant BJ, Freudenheim JL, Muti P, Browne RW, Drake JA, Klocke RA, Trevisan M (2001). The relation of serum levels of antioxidant vitamins C and E, retinol and carotenoids with pulmonary function in the general population.. American Journal of Respiratory and Critical Care Medicine.

[B24] Grievink L, de Waart FG, Schouten EG, Kok FJ (2000). Serum carotenoids, alpha-tocopherol, and lung function among Dutch elderly. American Journal of Respiratory and Critical Care Medicine.

[B25] MacNee W, Rahman I (2001). Is oxidative stress central to the pathogenesis of chronic obstructive pulmonary disease?. Trends Mol Med.

[B26] Steinmetz KA, Potter JD (1996). Vegetables, fruit, and cancer prevention: a review. Journal of the American Dietetic Association.

[B27] Peto R, Doll R, Buckley JD, Sporn MB (1981). Can dietary beta-carotene materially reduce human cancer rates?. Nature.

[B28] Moyad MA (2001). Results and lessons from clinical trials using dietary supplements for cancer: direct and indirect investigations. Seminars in Urologic Oncology.

[B29] Young AJ, Lowe GM (2001). Antioxidant and prooxidant properties of carotenoids. Archives of Biochemistry and Biophysics.

[B30] Armstrong BK, White E, Saracci R (1992). Principles of Exposure Measurement in Epidemiology.

[B31] Touloumi G, Pocock SJ, Babiker AG, Darbyshire JH (1999). Estimation and comparison of rates of change in longitudinal studies with informative drop-outs. Statistics in Medicine.

[B32] Littell RC, Rubin DB (1987). Statistical analysis with missing data.

[B33] Alfonso HS, Fritschi L, de Klerk NH, Ambrosini G, Beilby J, Oilsen N, Musk AW (2005). Plasma concentrations of retinol, carotene, and vitamin E and mortality in subjects with asbestosis in a cohort exposed to crocidolite in Wittenoom, Western Australia. J Occup Environ Med.

